# Angiotensin Converting-Enzyme Inhibitors, Angiotensin Receptor Blockers, and Calcium Channel Blockers Are Associated with Prolonged Vascular Access Patency in Uremic Patients Undergoing Hemodialysis

**DOI:** 10.1371/journal.pone.0166362

**Published:** 2016-11-10

**Authors:** Fu-An Chen, Chih-Chiang Chien, Yu-Wei Chen, Yu-Te Wu, Chih-Ching Lin

**Affiliations:** 1 School of Medicine, National Yang-Ming University, Taipei, Taiwan; 2 Department of Internal Medicine, National Yang-Ming University Hospital, Yi-Lan, Taiwan; 3 Department of Nephrology, Chi-Mei Medical Center, Tainan, Taiwan; 4 Division of Nephrology, Department of Internal Medicine, Taipei Veterans General Hospital, Taipei, Taiwan; Ehime University Graduate School of Medicine, JAPAN

## Abstract

**Background:**

Vascular access failure is a huge burden for patients undergoing hemodialysis. Many efforts have been made to maintain vascular access patency, including pharmacotherapy. Angiotensin converting enzyme inhibitor (ACE-I), angiotensin receptor blocker (ARB), and calcium channel blocker (CCB) are known for their antihypertensive and cardio-protective effects, however, their effects on long-term vascular access patency are still inconclusive.

**Design, setting, participants and measurements:**

We retrospectively enrolled patients commencing maintenance hemodialysis between January 1, 2000, and December 31, 2006 by using National Health Insurance Research Database in Taiwan. Primary patency was defined as the date of first arteriovenous fistula (AVF) or arteriovenous graft (AVG) creation to the time of access thrombosis or any intervention aimed to maintain or re-establish vascular access patency. Cox proportional hazards models were used to adjust the influences of patient characteristics, co-morbidities and medications.

**Results:**

Total 42244 patients were enrolled in this study, 37771 (89.4%) used AVF, 4473 (10.6%) used AVG as their first long term dialysis access. ACE-I, ARB, and CCB use were all associated with prolonged primary patency of AVF [hazard ratio (HR) 0.586, 95% confidence interval (CI) 0.557–0.616 for ACE-I use; HR 0.532, CI 0.508–0.556 for ARB use; HR 0.485, CI 0.470–0.501 for CCB use] and AVG (HR 0.557, CI 0.482–0.643 for ACE-I use, HR 0.536, CI 0.467–0.614 for ARB use, HR 0.482, CI 0.442–0.526 for CCB use).

**Conclusions:**

In our analysis, ACE-I, ARB, and CCB were strongly associated with prolonged primary patency of both AVF and AVG. Further prospective randomized studies are still warranted to prove the causality.

## Introduction

Vascular access is crucial for patients on maintenance hemodialysis. A functional long-term vascular access is associated better life quality [1], less mortality [2–4] and hospitalization [5]. However, vascular access occlusion is still a major cause of hospitalization in patients undergoing hemodialysis [6]. And the cost for vascular access failure is still high in recent years.

Many strategies have been surveyed in order to prolong vascular access patency, including meticulous pre-operative planning [7], newer design of vascular access [8], stent implantation [9,10], far infrared therapy [11], and pharmacotherapy [12–16]. Among pharmacotherapy, some cardioprotective antihypertensive agents have drawn attention recently, including angiotensin converting enzyme inhibitor (ACE-I), angiotensin receptor blocker (ARB) and calcium channel blocker (CCB). Theoretically, ACE-I, ARB and CCB could increase vascular access patency through inhibiting venous neointimal hyperplasia, an important mechanism of arteriovenous fistula (AVF) and arteriovenous graft (AVG) failure [17–21]. However, the results of these drugs on clinical studies were still controversial [12,13,16,22–24]. The aim of this study is to evaluate whether ACE-I, ARB, and CCB could have impact on long-term vascular access patency.

## Materials and Methods

### Database

The National Health Insurance (NHI) program has provided compulsory universal health insurance in Taiwan since 1995. With the exception of prison inmates, all citizens have been enrolled in the program. All contracted medical institutions must submit standard computerized claim documents for medical expenses. Patients with End stage renal disease (ESRD) are eligible for any type of renal replacement therapy free of any charge; all maintenance dialysis patients are covered by NHI.

Data were obtained from the National Health Insurance Research Database (NHIRD) [Bureau of National Health Insurance. Available at: www.doh.gov.tw/statistic/index.htm [In Chinese] (accessed September 25, 2011); http://www.doh.gov.tw/EN2006/index_EN.aspx [In English]] and released for research by the Taiwan National Health Research Institute. The NHIRD covers nearly all (99%) inpatient and outpatient medical benefit claims for Taiwan’s 23 million residents, is one of the largest and most comprehensive databases in the world, and has been used extensively in various studies. Patient identification numbers, gender, birthday, dates of admission and discharge, medical institutions providing the services, the ICD-9-CM (International Classification of Diseases, 9th Revision, Clinical Modification) diagnostic and procedure codes, and outcomes are encrypted. We used the NHIRD for ambulatory care claims, all inpatient claims, and the updated registry for beneficiaries for this study. All datasets can be interlinked through each individual’s unique personal identification number.

### Patient selection and definition

Incident adult ESRD patients (≥18 years old) who began maintenance hemodialysis between January 1, 2000, and December 31, 2006 were included in this study. ESRD patients on maintenance hemodialysis were defined as having undergone hemodialysis for more than 90 days. All incident patients with first payment and operation code for AVF (procedure code 69032C) or AVG (procedure code 69034C) between January 1, 1998, and December 31, 2008 were identified to analyze. A total of 42244 incident dialysis patients were analyzed in this study.

The definition of primary patency was the time from the first reported date of vascular access creation to the date of access thrombosis or any intervention aimed to maintain or re-establish vascular access patency. Patients were followed during the period of primary patency. Patient who died or discontinuing dialysis, or whose vascular access remained patent during hemodialysis after December 31, 2008 were censored. Vascular access dysfunction was identified based on the diagnostic code for vascular thrombosis (ICD-9-CM 996.73, other complications due to renal dialysis device implant and graft) and operative procedures for vascular thrombotic occlusion (thrombectomy, procedure code 38.0; reconstruction of access, procedure code, 69032C; embolectomy, arterial, 69001B; embolectomy, arterial catheter, 69002B; thrombectomy, venous, 69003B) at a subsequent admission or outpatient visit. Vascular access related intervention [percutaneous transluminal angiography (PTA), revision or removal of vascular access] were identify either by NHI' s procedure code or by disease code.

### Ascertaining the demographic and comorbid variables

We linked to the diagnostic codes through the inpatient and outpatient claims databases of the NHI. We included patient demographics, and baseline comorbidities. Baseline comorbidities—including diabetes mellitus (DM), hypertension (HTN), coronary artery disease (CAD), ischemic cerebrovascular disease, deep venous thrombosis (DVT), peripheral artery disease (PAD), dyslipidemia, hyperuricemia, chronic liver disease—were analyzed. In addition, the use of ACE-I, ARB, or CCB for more than 3 months after creation of AVF or AVG was also identified by the codes specified for those drugs. The drugs evaluated in the study were listed in Table 1.

**Table 1:** Drugs included in the study.

Angiotensin converting enzyme inhibitor	Angiotensin receptor blocker
Benazepril	Candesartan
Enalapril	Losartan
Lisinopril	Irbesartan
Quinapril	Valsartan
Captopril	Olmesartan
Fosinopril	Calcium channel blocker
Ramipril	Amlodipine
Verapamil	Felodipine
Cilazapril	Nifedipine
	Verapamil
	Diltiazem
	Isradipine
	Nicardipine

### Statistical analysis

Parametric Pearson’s chi square test is utilized to compare each variable in the groups of patients with using AVF and AVG. Age was entered as a categorical variable (18 to 44, 45 to 64, and 65 years or older). Significance was set at *p* < 0.05. The cumulative incidences of vascular dysfunction after vascular access creation were calculated using the Kaplan-Meier method. The log rank test was used to analyze significance. Cox proportional hazards models were used to identify the risk factors of vascular access dysfunction after vascular access creation. Hazard ratios (HR) and 95% confidence intervals (CI) were derived from Cox proportional hazards models. Cox models met the assumption of proportionality of risks. To adjust for potential confounding in the relationship between variables and the vascular access dysfunction, multivariate analyses were used. All statistical operations were performed using the Statistical Package for Social Sciences for Windows 17.0 (SPSS Inc; Chicago, IL, USA).

## Results

### Patient characteristics

**Fig 1:**
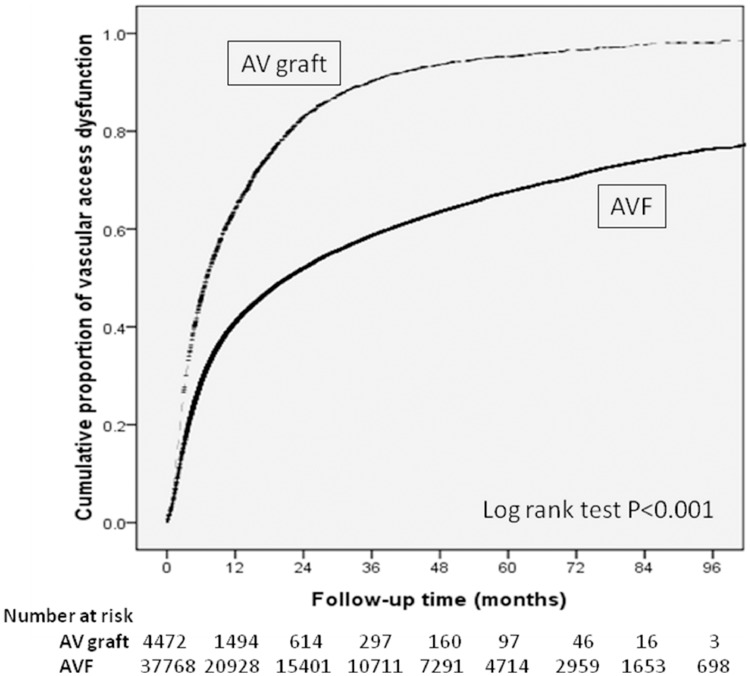
Cumulative incidence of vascular access dysfunction.

Total 42244 patients were enrolled in this study. 37771 (89.4%) used AVF, 4473 (10.6%) used AVG as their first long term dialysis access (Table 2). Patients using AVF were male predominant, older, and they had lower prevalence of diabetes mellitus (*p* < 0.001), coronary artery disease (*p* < 0.001), ischemic cerebrovascular disease (*p* < 0.001), peripheral artery disease (*p* = 0.005), and deep vein thrombosis (*p* = 0.039). AVF group also had higher rate of using renin-angiotensin blockage and CCB. Compared with patients using AVF, those using AVG had higher frequency of creation of vascular access before dialysis (75.9% V.S 68.8%). The cumulative patency of primary AVG and AVF was 35.9% and 59.2% at the end of year 1, but only 4.7% and 32.5% at the end of year 5 respectively. The incidence of primary patency was significantly higher in AVF group both in univariate and multivariate analysis (Fig 1).

**Table 2:** Patient characteristics and association with using arteriovenous fistula and arteriovenous graft among end-stage renal disease hemodialysis patients.

	Patients Using an AVF (n = 37771)		Patients Using an AVG (n = 4473)		*P* value
	n	(%)	n	(%)	
Sex					<0.001
Female	18327	(86.6)	2841	(13.4)	
Male	19444	(92.3)	1632	(7.7)	
Age (years)					<0.001
18–44	5058	(94.5)	296	(5.5)	
45–64	17446	(91.3)	1671	(8.7)	
≥ 65	15267	(85.9)	2506	(14.1)	
Time of vascular access creation					<0.001
After initiating dialysis	13214	(91.6)	1209	(8.4)	
0–1 month before dialysis	13323	(87.8)	1843	(12.2)	
≥ 1 month before dialysis	11234	(88.8)	1421	(11.2)	
Diabetic mellitus					<0.001
No	18943	(90.6)	1954	(9.4)	
Yes	18828	(88.2)	2519	(11.8)	
Hypertension					0.232
No	7144	(89.8)	813	(10.2)	
Yes	30627	(89.3)	3660	(10.7)	
Coronary artery disease					<0.001
No	29122	(90.1)	3204	(9.9)	
Yes	8649	(87.2)	1269	(12.8)	
Ischemic cerebrovascular disease					<0.001
No	35791	(89.6)	4139	(10.4)	
Yes	1980	(85.6)	334	(14.4)	
Deep venous thrombosis					0.039
No	37533	(89.4)	4433	(10.6)	
Yes	238	(85.6)	40	(14.4)	
Peripheral artery disease					0.005
No	36654	(89.5)	4307	(10.5)	
Yes	1117	(87.1)	166	(12.9)	
Dyslipidemia					0.427
No	31310	(89.4)	3729	(10.6)	
Yes	6461	(89.7)	744	(10.3)	
Hyperuricemia					0.129
No	32371	(89.3)	3871	(10.7)	
Yes	5400	(90.0)	602	(10)	
Chronic liver disease					0.131
No	34431	(89.5)	4047	(10.5)	
Yes	3340	(88.7)	426	(11.3)	
Drug Usage (%)					
ACE-I	10.7%		6.2%		<0.001
ARB	15.0%		7.1%		<0.001
CCB	32.2%		20.6%		<0.001

AVF: arteriovenous fistula; AVG: arteriovenous graft; ACE-I: angiotensin converting enzyme inhibitor; ARB: angiotensin receptor blocker; CCB: calcium channel blocker.

### Patient characteristics associated with AVF and AVG dysfunction

Tables 3 and 4 showed the factors associated with vascular access dysfunction. In patients with AVF, patient characteristics relating to more access dysfunction in multivariate analysis were female sex (HR 0.964, CI 0.939–0.990), age (HR 1.177, CI 1.129–1.228 in those aged 45–64, HR 1.329, CI 1.272–1.387 in those aged ≥ 65, comparing to reference group aged 18–44), diabetes mellitus (HR 1.264, CI 1.229–1.300), hypertension (HR 1.149, CI 1.110–1.189), coronary artery disease (HR 1.109, CI 1.074–1.144), deep venous thrombosis (HR 1.267, CI 1.087–1.476), peripheral artery disease (HR 1.195, CI 1.111–1.285), dyslipidemia (HR 1.101, CI 1.063–1.140). Early AVF creation was associated with less AVF dysfunction. Comparing to reference group who received AVF creation after commencing hemodialysis, there’s a HR 0.969, CI 0.940–0.999 in group whose AVF created 0-1 month before, and a HR 0.948, CI 0.917–0.979 in group whose AVF created more than 1 month before commencing hemodialysis.

**Table 3:** Risk factors for arteriovenous fistula dysfunction among maintenance hemodialysis patients.

Covariate	Univariate analysis	Multivariate analysis
	HR (95% CI)	HR (95% CI)
Sex (Male vs. Female)	0.939 (0.916–0.963)*	0.964 (0.939–0.99)*
Age at initiation of Dialysis		
18–44 (Reference)	1	1
45–64	1.251 (1.201–1.304)*	1.177 (1.129–1.228)*
≥ 65	1.512 (1.451–1.577)*	1.329 (1.272–1.387)*
Time of vascular access creation		
After initiating dialysis (Reference)	1	1
0–1 month before dialysis	0.951 (0.922–0.980)*	0.969 (0.940–0.999)*
More than 1 month before dialysis	0.839 (0.812–0.866)*	0.948 (0.917–0.979)*
Comorbidities		
Diabetic Mellitus (yes vs. no)	1.228 (1.197–1.260)*	1.264 (1.229–1.300)*
Hypertension (yes vs. no)	1.083 (1.048–1.119)*	1.149 (1.110–1.189)*
Coronary Artery Disease (yes vs. no)	1.175 (1.140–1.211)*	1.109 (1.074–1.144)*
Ischemic Cerebrovascular disease (yes vs. no)	1.142 (1.079–1.209)*	1.045 (0.986–1.107)
Deep venous thrombosis (yes vs. no)	1.410 (1.210–1.642)*	1.267 (1.087–1.476)*
Peripheral artery disease (yes vs. no)	1.266 (1.178–1.361)*	1.195 (1.111–1.285)*
Dyslipidemia (yes vs. no)	1.083 (1.047–1.120)*	1.101 (1.063–1.140)*
Hyperuricemia (yes vs. no)	1.005 (0.696–1.042)	1.031 (0.993–1.071)
Chronic liver disease (yes vs. no)	0.950 (0.907–0.995)*	1.003 (0.970–1.036)
Drug Usage		
ACE-I (yes vs. no)	0.428 (0.407–0.449)*	0.586 (0.557–0.616)*
ARB (yes vs. no)	0.386(0.370–0.404)*	0.532 (0.508–0.556)*
CCB (yes vs. no)	0.405 (0.393–0.417)*	0.485 (0.470–0.501)*

ACE-I: angiotensin converting enzyme inhibitor; ARB: angiotensin receptor blocker; CCB: calcium channel blocker.

*indicates a significant difference (*p*<0.05).

**Table 4:** Risk factors for arteriovenous graft dysfunction among maintenance hemodialysis patients.

Covariate	Univariate analysis	Multivariate analysis
	HR (95% CI)	HR (95% CI)
Sex (Male v Female)	1.010 (0.947–1.078)	0.989 (0.925–1.056)
Age at initiation of Dialysis		
18–44 (Reference)	1	1
45–64	1.056 (0.927–1.202)	1.126 (0.988–1.285)
≥ 65	1.137 (1.002–1.290)*	1.207 (1.061–1.373)*
Time of vascular access creation		
After initiating dialysis (Reference)	1	1
0–1 month before dialysis	0.923 (0.855–0.997)*	0.966 (0.894–1.044)
More than 1 month before dialysis	0.766 (0.706–0.831)*	0.857 (0.789–0.931)*
Comorbidities		
Diabetic Mellitus (yes vs. no)	1.072 (1.006–1.141)*	1.143 (1.068–1.223)*
Hypertension (yes vs. no)	0.974 (0.898–1.055)	1.064 (0.977–1.159)
Coronary Artery Disease (yes vs. no)	1.000 (0.933–1.072)	1.018 (0.947–1.093)*
Ischemic Cerebrovascular disease (yes vs. no)	1.079 (0.957–1.217)	1.073 (0.950–1.212)
Deep venous thrombosis (yes vs. no)	1.339 (0.973–1.743)	1.374 (0.998–1.863)
Peripheral artery disease (yes vs. no)	1.058 (0.896–1.480)	1.047 (0.886–1.237)
Dyslipidemia (yes vs. no)	0.979 (0.900–1.065)	1.038 (0.952–1.132)
Hyperuricemia (yes vs. no)	1.024 (0.935–1.121)	1.081 (0.984–1.187)
Chronic liver disease (yes vs. no)	0.954 (0.857–1.061)	1.010 (0.931–1.096)
Drug Usage		
ACE-I (yes vs. no)	0.407 (0.354–0.468)*	0.557 (0.482–0.643)*
ARB (yes vs. no)	0.396 (0.347–0.452)*	0.536 (0.467–0.614)*
CCB (yes vs. no)	0.400 (0.369–0.434)*	0.482 (0.442–0.526)*

ACE-I: angiotensin converting enzyme inhibitor; ARB: angiotensin receptor blocker; CCB: calcium channel blocker.

*indicates a significant difference (*p*<0.05).

Patient characteristics associated with more AVG dysfunction in multivariate analysis were older age (HR1.207, CI 1.061–1.373 in group aged ≥ 65, but no significance in those aged 45–64, comparing with reference group aged 18–44), diabetes mellitus (HR 1.143, CI 1.068–1.223), and coronary artery disease (HR 1.018, CI 0.947–1.093). AVG created more than 1 month before hemodialysis was associated with less AVG dysfunction comparing with AVG created after hemodialysis(HR 0.857, CI 0.789–0.931), but there’s no difference in AVG created 0–1 month before HD comparing with the reference group(HR 0.966, CI 0.894–1.044).

### Medications and AVF and AVG dysfunction

**Fig 2:**
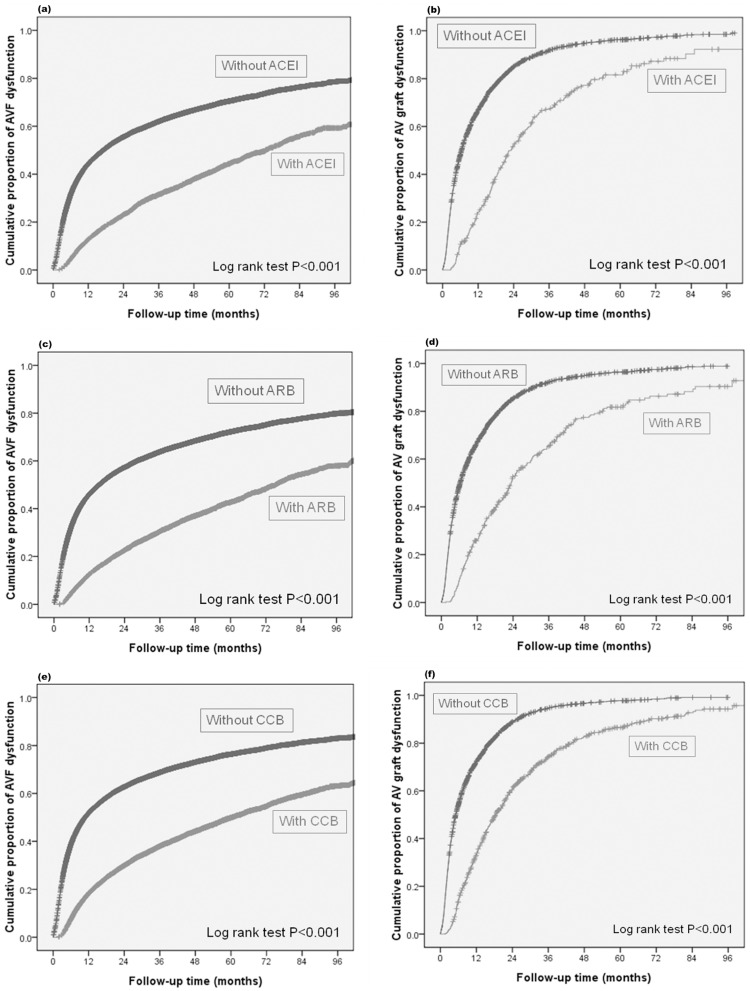
Cumulative incidence of vascular access dysfunction stratified by drugs. (a) ACEI & AVF (b) ACEI & AVG (c) ARB & AVF (d) ARB & AVG (e) CCB & AVF (f) CCB & AVG.

Fig 2 showed Kaplan-Meier curve of medication use and cumulative incidences of vascular access dysfunction. All three kinds of medications were related to less AVF dysfunction in multivariate analysis, including ACE-I (HR 0.586, CI 0.557–0.616), ARB (HR 0.532, CI 0.508–0.556) and CCB (HR 0.485, CI 0.470–0.501). These medications were also associated with less AVG dysfunction by multivariate analysis [ACE-I (HR 0.557, CI 0.482–0.643), ARB (HR 0.536, CI 0.467–0.614), CCB (HR 0.482, CI 0.442–0.526)].

## Discussion

The objective of the study was to evaluate the relationship between anti-hypertensive drugs and primary patency of long-term vascular access. Our analysis revealed that ACE-I, ARB, and CCB were all associated with decreased risk of AVF and AVG dysfunction. For AVF, the relative risk reduction in ACE-I, ARB, and CCB user were 41%, 47%, and 51%. For AVG, the relative risk reduction associated with ACE-I, ARB, and CCB user were 44%, 46%, and 52%.

There were researches aimed for ACE-I and vascular access patency. Gradzki [23] et al. found that ACE-I was associated with higher AVG patency. Saran [13] et al. also reported that ACE-I was related to prolonged secondary patency of AVF. Our study also showed ACE-I was associated with prolonged primary patency in both AVG and AVG groups.

The association between vascular access patency and use of ARB is controversial. Jackson et al reported that ARB prolongs primary patency in both AVF and AVG groups [12], whereas the DOPPS study [13] showed that there’s no relationship between ARB and vascular access patency, neither in AVF or AVG. However, there is a relatively low rate of ARB use in the DOPPS population (3.8% in AVG group, and 4.1% in AVF group). The rates of using ARB in our AVF and AVG groups were 15.0% and 7.1%, respectively. This turned out to be about 5666 and 318 patients using ARB in our AVF and AVG group, which may increase the statistical power. Moreover, the subjects included in our study were somewhat different from those in DOPPS:Our study focused on the patients whose long-term vascular access was first created, most of them were incident ESRD patients; Whereas the DOPPS enrolled all eligible patients with newly created vascular access, including those with previous permanent accesses. The rate of incident ESRD patients in AVF and AVG groups in DOPPS study were 46.4% and 32.5%, respectively. DOPPS revealed higher numbers of previous access is a risk factor for vascular access failure, which may indicate that long-term dialysis provokes atherosclerosis and arteriosclerosis and overwhelms the effect of vascular access protection of ARB.

Our study also revealed a significant relationship between CCB and prolongation of primary patency in both AVF and AVG group. Animal [18] and human [25] studies had proved that CCB could inhibit vascular neointimal hyperplasia, which is a major characteristic of vascular access stenosis. In clinical studies, CCB was associated with increasing maturation rate of newly created AVF [16]. Doi et al also reported that CCB significantly decreased restenosis rate of AVF after percutaneous transluminal angioplasty [24]. Although this effect was not significant in their AVG group, which may be ascribed to a relatively small number of subjects, there was a trend toward benefit. DOPPS study showed that CCB was associated with increased primary patency rate of AVG, but not AVF.

Our study had several limitations. First, the definition of users of the specified anti-hypertensive agent were those who had used this specified medicine for more than 3 months after the creation of their first AVF or AVG. Some patients who used the medicine for a period shorter than 3 months would be classified as non-users, and those who classified as users may have poor drug compliance. These may weaken the relationship between these medications and vascular access patency in the analysis. Second, this is a retrospective observational study, we could find the relationship between medications and vascular access patency, but the causality cannot be proved. Third, the study is based on NHI claim database, thus some factors, including patient’s laboratory data, smoking history, the control of blood pressure, severity of comorbid diseases, and the location of AVFs or AVGs, could not be identified. The control of blood pressure is of especial importance because ACEI, ARB, and CCB are anti-hypertensive agents. Although ACEI, ARB, and CCB were still significantly associated with increased primary patency after adjusting hypertension in multivariate analysis, poor control of blood pressure may still interfere with the result. Fourth, the original indications of these medications should be mostly used for control of blood pressure rather than prevention of vascular access dysfunction. Whether the study results could be extrapolated to normotensive or hypotensive patients still remains to be determined.

There were also some notable points of our study. Our claim database derived from a nationwide, universal coverage health system, which diminished the possibility of selection bias and provided a considerable amount of eligible subjects. Besides, although randomized, placebo-controlled trials provide evidence of highest level for medical practice. Sometimes it might be limited by ethical problems. In our study, medications such as ACE-I, ARB, and CCB had been proved for its cardioprotective effect in patients undergoing dialysis, which makes it difficult to prohibit their use in a randomized controlled study. Our database study in nature obviated this problem and could provide information for the design of further randomized controlled trials.

In conclusion, our study suggests that ACE-I, ARB, and CCB are associated with increased primary patency of first created AVF and AVG. This large observational, nationwide claim database study may be helpful in clinical practice for patient with newly created vascular access and also serves as a guide for further prospective, randomized, controlled studies.
